# Inflammation: Is It a Healer, Confounder, or a Promoter of Cardiometabolic Risks?

**DOI:** 10.3390/biom14080948

**Published:** 2024-08-06

**Authors:** Amit R. Tate, Gundu H. R. Rao

**Affiliations:** 1 South Asian Society on Atherosclerosis and Thrombosis (SASAT), Minneapolis, MN 55455, USA; amitrtate@gmail.com; 2 Laboratory Medicine, and Pathology, Thrombosis Research, Lillehei Heart Institute, University of Minnesota, Minneapolis, MN 55455, USA

**Keywords:** inflammation, hypertension, obesity, diabetes, vascular disease

## Abstract

Inflammation is the body’s non-specific response to injury or infection. It is a natural defense mechanism that helps to maintain homeostasis and promotes tissue repair. However, excessive inflammation can lead to cellular, tissue, or organ dysfunction, as well as contribute to the development of acute vascular events and diseases like Crohn’s disease, psoriasis, obesity, diabetes, and cancer. The initial response to injury involves the activation of platelets and coagulation mechanisms to stop bleeding. This is followed by the recruitment of immune cells and the release of cytokines to promote tissue repair. Over time, the injured tissue undergoes remodeling and returns to its pre-injury state. Inflammation is characterized by the activation of inflammatory signaling pathways involving cytokines, chemokines, and growth factors. Mast cells play a role in initiating inflammatory responses. Pattern recognition receptors (PRRs) such as Toll-like receptors (TLRs) and nucleotide-binding domain (NOD)-like receptors (NLRs) are involved in the activation of these inflammatory pathways. Inflammasomes, which are cytoplasmic complexes, also contribute to inflammation by activating cytokines. Inflammation can also be triggered by factors like dietary components and the composition of the gut microbiota. Dysregulation of the gut microbiome can lead to excessive inflammation and contribute to diseases like atherosclerosis and irritable bowel syndrome (IBS). The immune system and gut-associated lymphoid tissue (GALT) play crucial roles in the inflammatory response and the development of conditions like colorectal cancer. Anti-inflammatory therapy can play a significant role in reducing or inducing the remission of inflammatory diseases such as Crohn’s disease and ulcerative colitis. The fetal origin of adult diseases theory suggests that conditions during fetal development, such as low birth weight and maternal obesity, can influence the risk of cardiometabolic diseases later in life. All of the known risk factors associated with cardiometabolic diseases such as hypertension, excess weight, obesity, type-2 diabetes, and vascular diseases are accompanied by chronic low-grade inflammation. Inflammation seems to have a role in precipitating even acute vascular events such as heart attacks and stroke. Common markers of inflammation associated with cardiometabolic disease include interleukin (IL)-1β, IL-6, tumor necrosis factor (TNF-α), C-reactive protein (CRP), and soluble TNF receptors such as sTNFR1 and sTNFR2. These markers serve as indicators of systemic inflammation. However, these markers are not disease-specific but provide an insight into the overall chronic inflammatory status. In fact, inflammation has been identified as a potential target for future treatments to reduce or reverse the risk of atherosclerosis-related complications. The regulation of inflammation is complex, and further research is needed to better understand its mechanisms and develop strategies for managing inflammatory disorders. In summary, inflammation is a natural response to injury or infection, but excessive or prolonged inflammation can lead to the progression of various diseases. Understanding the underlying mechanisms of inflammation is important for developing treatments and preventive measures for inflammatory disorders.

## 1. Introduction

The body’s non-specific response to injury or infection is called inflammation, part of the normal, delicate physiological response of homeostasis [1]. Excessive inflammation can lead to cellular, tissue, or organ dysfunction, destruction, or even the promotion of acute vascular events as well as malignancies [2]. Inflammation is the first natural response to any injury, external or internal. Injury could be at the level of organs, tissues, cells, or at the molecular level [3]. Depending upon the nature of the injury, the local responses will be immediate and continuous, until there is a full restoration of homeostasis. Failure to terminate this natural defense mechanism could result in the initiation and progression of metabolic diseases, immune-compromised diseases, and diseases of the skin, lungs, various organ systems, and cells [4]. If we look at a typical response to injury, assuming the injury results in bleeding, then it starts first with the primary purpose of arresting bleeding by activating platelet and coagulation mechanisms. The tissue repair process continues with the recruitment of platelets to the area of injury, recruitment of leukocytes, macrophages, and release of cytokines. The wound-healing process continues at the cellular level with re-epithelialization, angiogenesis, fibroplasia, and wound contraction [4,5]. Remodeling takes place over weeks or months during which the dermis regrows, followed by a reduction in matrix proteins and then a return to the preinjury phenotype. Not all injuries lead to bleeding; hence, we cannot propose this sequalae of events as the common remedial measures for all types of injuries. Inflammation seems to be a common feature of metabolic disturbances, whether it is acute events leading to heart attack and stroke, or chronic metabolic disorders such as Crohn’s disease, irritable bowel syndrome, dermatitis, psoriasis, eczema, or for that matter noncommunicable diseases such as various respiratory diseases, metabolic disease like hypertension, excess weight, obesity, diabetes, vascular disease, stroke, and cancer [6,7,8,9,10].

Inflammation, whether it is a healer of tissue injury or a confounder of various disease processes or a promoter of metabolic risks, shares some common features [11,12,13,14,15,16,17,18,19,20,21,22,23,24,25]. The inflammation theory of disease is the growing realization that chronic inflammation is a crucial factor for the development of many diseases. This theory proposes that persistent inflammatory mediators, such as cytokines and chemokines, can induce long-term damage and dysfunction when present in inflamed areas [8]. As far as the cardiometabolic diseases are concerned, the following markers of inflammation have been reported; IL-1β, IL-6, TNFα, C-reactive protein (CRP), and the soluble TNF receptors [12]. Inflammation, therefore, is an immune response of the human body and by and large, the major signaling pathways of the inflammatory response are cytokines like interleukins (ILs), chemokines, interferons (INF), tumor necrosis factors (TNF-α), growth factors, and colony-stimulating factors [18]. While CRP and Il-6 are often correlated in clinical studies, it is important to consider the specific relationships between CRP, Il-6, and other cytokines, as the correlations may differ depending on the clinical setting. Yet another area which is complementary is mitochondrial dysfunction leading to metabolic diseases. They are the central hubs in energy metabolism, immunity, and signal transduction.

Mast cells, which reside in connective tissue matrices and on epithelial cell surfaces, seem to be effector cells that imitate inflammatory responses [1]. According to researchers, irrespective of the type of injury, pathogen, damaged cell, allergen, or toxic compound, the activation of the inflammatory cells triggers inflammatory signaling pathways, in general by the NF-kB, MAPK, and JAK-STAT pathways [5,6,7,8,9]. They all seem to have a common mechanism, which includes the involvement of cell-surface pattern recognition receptors (PRRs), which recognize the detrimental stimuli, initiate the activation of inflammatory pathways, promote the release of inflammatory markers, and recruit inflammatory cells. This family of receptors includes Toll-like receptors (TLRs), retinoic acid-inducible gene (RIG)-I-like receptors (RLRs), and nucleotide-binding domain (NOD)-like receptors (NLRs).

Inflammasomes are cytoplasmic multiprotein complexes that contain a sensor protein, inflammatory caspases, and an adapter protein. The activation of TLRs, RLRs, and NLRs initiates signaling to promote the upregulation of proinflammatory mediators, whereas the activation of NLRs, such as interferon inducible protein (AIM2), promote the formation and activation of inflammasomes (20). Inflammasomes are the cellular sensors that convey the cellular stress as well as pathogen presence to the immune system by activating inflammatory caspases and cytokines 1L-1β [21]. It is not very clear at this point how exactly various injury signals initiate inflammation. Since the degree and intensity of the response to injury varies considerably in various situations, such as from an insect bite to a COVID-19 mediated cytokine storm, there must be complex signaling mechanisms that modulate the injury–inflammation signaling process [22,23,24]. It is important to note that for an effective response against a viral pathogen such as COVID-19, T-helper (Th) cells regulate the adaptive immune system by cytokine production. In the case of COVID-19 infection, it has been noted that TH_2_ responses are promoted, accompanied by the overexpression of Th_2_-derived cytokines, IL-4, IL-5, IL-13 [23]. It is believed that immune dysregulation is associated with a poorer outcome of COVID-19 pneumonia. A clinical study performed in multiple sites with anti-inflammatory agents failed to speed up recovery, although they may have prevented excess deaths from so-called “cytokine storms” [26,27,28].

Certain gut bacteria produce butyrate which exerts anti-inflammatory effects in the intestinal mucosa. The depletion of such microbes or dysfunction of butyrate production may cause the leakage of microbial toxins such as LS which bind to TLRs and trigger inflammation [28,29,30,31,32]. Gut microbiota have been implicated in subclinical atherosclerosis. In a recent study, Swedish researchers found evidence of an association between a gut microbiota composition characterized by an increased abundance of *Streptococcus* spp found in oral cavity, with coronary atherosclerosis and systemic inflammation [2]. Between 500 and 1000 species of microbes colonize the large intestine of humans. Dysfunctional regulation of this huge gut microbiome leads to excessive polarization towards the T-helper-1 (TH_1_) phenotype and the initiation of irritable bowel syndrome (IBS) [10,11]. According to immunologists, patients with Crohn’s disease (CD) or ulcerative colitis (UC) have a 12 to 20% risk of developing colorectal cancer (CRC). When considering the role of the gut microbiome, we should understand that 70% of the immune system is localized in the gastrointestinal tract. Added to this, the system is very close to the intrabdominal white adipose tissue (WAT), which in obese individuals is populated by a large number of inflammatory macrophages. Gut-associated lymphoid tissue (GALT) seems to be dysfunctional and plays a role in impaired host defense in colorectal cancer patients. It is believed that crosstalk between GALT and WAT may play a role in shaping the inflammatory responses that lead to CRC [29,30,31].

We mentioned at the very outset that the body’s non-specific response to injury or infection is called inflammation. It is more like a biological alarm system, which puts a variety of cellular and molecular mechanisms into motion to achieve normal homeostasis. Having said that, we have to inform readers that it is not always favorable, and we are not in a position to explain when the normal molecular signaling changes direction and becomes harmful to normal physiology and function. When discussing risks for cardiometabolic diseases, the earliest risk recorded by epidemiologists is the fetal origin of adult diseases [32]. The Barker hypothesis suggests that intrauterine growth retardation, low birth weight, and premature birth have a causal relationship with the origins of hypertension, coronary artery disease, and non-insulin-dependent diabetes in middle age [32]. Apart from intrauterine nutrition, maternal nutrition and maternal obesity should be addressed when discussing this early origin of adult diseases [33,34]. Recently, researchers at the Children’s National Hospital, Washington DC, have demonstrated a role of maternal obesity as a possible contributor for the so-called “thin fat baby syndrome” described by the Pune Maternal Nutrition Study Group [35,36,37,38,39]. Furthermore, the same group have also described high levels of circulating proinflammatory cytokines and leptin in urban but not rural Indians, a potential explanation for the increased risk of diabetes and coronary heart disease [39]. In this overview, we briefly discuss the role of inflammation as healer of tissue injury, a confounder of various disease states including cardiometabolic diseases.

## 2. Inflammation and Wound Healing

**Figure 1:**
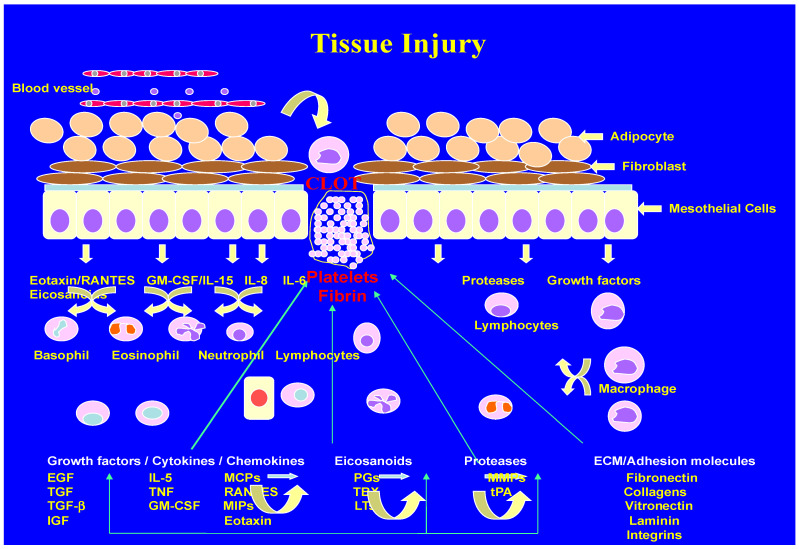
Schematic representation of wound-healing mechanisms. (University of Minnesota Artists, Personal Collection).

In general, wound healing involves three basic mechanisms: contraction, connective tissue matrix deposition, and epithelialization [40,41,42,43]. In technical terms the sequence of events would be hemostasias, inflammation, proliferation, and remodeling. Shown in the Figure 1 is a schematic representation of such a sequence of events, wherein platelets as the first responders identify the injury and initiate a clot to arrest the bleeding. The complex process of wound healing involves a variety of specialized cells, such as platelets, macrophages, fibroblasts, epithelial cells, and endothelial cells. As far as molecular mechanisms are concerned, the healing process is influenced by a variety of proteins, glycoproteins, cytokines, chemokines, growth factors, inhibitors, and specific receptors for signal transmission and fine-tuning of the healing process. When an injury occurs to blood vessels and exposes the extracellular matrix components, platelets become activated and initiate adhesion, aggregate formation, and the secretion of granule contents including adhesion molecules, vasoconstrictors, and growth factors. Key glycoproteins released include fibrinogen, fibronectin, thrombospondin, and von Willebrand factor. Activated platelets also express acidic lipids on their membranes, promote the formation of prothrombinase, and express tissue factor [40].

Immediately after the injury, following the degranulation of activated platelets, the second responder inflammation activates the innate immune system, which includes the recruitment of inflammatory cells from the circulating blood. Local immune cells such as macrophages are activated in response to tissue injury. Damage-associated molecular pattern recognition molecules (DAMPs) and other responders for inflammatory stimulus are induced into action. Furthermore, the hypoxic environment of the wound also promotes inflammation, as hypoxia stimulates numerous cell types. Since the wound-healing process occurs in phases, leukocytes which are abundant in the circulation infiltrate the wound quickly and recruit monocytes, which differentiate into mature macrophages. These events are followed by the infiltration of mast cells and at the late stages of wound repair, which is a lengthy phase, T lymphocytes also appear in the wounds. No definitive role could be assigned to inflammation for the wound-repair phase, it is believed that the inflammatory phases have a profound effect on the final outcome of wound healing [40,41,42,43,44,45].

In a one-of-a-kind study at the RV Dental College, Bengaluru, India, we demonstrated a yet another role for platelets in wound healing and bone regeneration. We used platelet-rich fibrin gels prepared with calcium-induced gel formation, to promote wound healing and bone regeneration post molar extraction in 22 volunteer patients. Post extraction of the tooth, the sockets were filled with autologous platelet-rich fibrin gel. Patients were recalled at regular intervals of the first, second, and sixth months. Radiographs were taken and converted to a digital format connected to a computer. Using proprietary software (Corel Draw Software 2013) analytics, the progression of wound healing and bone regeneration in control subjects and treatment groups were computed. In this study, we observed accelerated soft tissue healing in the treatment groups, as well as faster and better bone regeneration, compared to the untreated control subjects [45].

## 3. Inflammation as a Promoter of Cardiometabolic Diseases

Despite the fact that cardiovascular disease (CVD) is ranked as the number one killer—based on the observation that in 2019, it was the cause for approximately 18 million deaths worldwide—little is known about the molecular mechanisms that drive the progression of the disease, leading to occlusive arterial events. Prevalent cases of total CVD nearly doubled from 271 in 1990 to 523 million in 2019, and the number of CVD deaths steadily increased from 12.2 million in 1990 to 18.6 million in 2019 [46]. The sequalae of events that initiate and promote the progression of the cardiovascular disease include oxidative stress, inflammation, altered blood glucose, blood lipid metabolism, impaired glucose tolerance, insulin resistance, endothelial dysfunction, subclinical atherosclerosis, narrowing of the artery development of atherosclerotic plaques, and the activation of pathways that lead to thrombotic conditions [47,48,49,50,51,52,53,54,55,56,57,58].

Inflammation seems to be a common confounder of all the cardiometabolic risks listed above [59]. Despite this knowledge, clinicians concentrate mostly on the management of modifiable risk factors such as blood pressure, blood lipids, blood glucose, and associated disease conditions such as hypertension, excess weight, obesity, and type-2 diabetes. Furthermore, we do not know exactly how inflammation drives the progression of these CVD risk factors, including occlusive vascular events such as heart attacks and stroke. In this overview, we briefly discuss inflammation and its major role, if any, in promoting one or more of the sequalae of events associated with the development and progression of metabolic risk factors or metabolic diseases.

## 4. Oxidative Stress and Reactive Nitrogen Intermediates (RNIs) in Inflammation

Oxidative stress generates reactive oxygen species (ROS) and contribute to the initiation and progression of the pathogenesis of various diseases. Oxidative stress in normal situations is a balance between the production of reactive oxygen species and their removal by various protective mechanisms. Glutathione is a major antioxidant that can alleviate this condition by the removal of ROS [60]. Researchers have demonstrated that exogenously administered glutathione is able to protect RAW 264.7 cells against oxidative stress and induce mitochondria-mediated apoptosis, along with the activity of the Nrf2/HO-1 signaling pathway. Under oxidative stress, ROS are able to mediate endothelial dysfunction and vascular abnormalities by disrupting the vasoprotective nitric oxide (NO) signaling pathway [55]. Although we are discussing the role of oxidative stress, it is worth noting that nitric oxide modulates an anti-inflammatory feedback loop via downstream reactive nitrogen intermediates (NRIs) which are toxic to microbes as well as host cells [61]. Oxidized LDL particles (ox-LDLs) generated by the mediation of ROS, seem to be a major trigger for atheroma plaque formation and development, because these modified LDLs are significantly more potent than the native LDLs in promoting endothelial dysfunction, one of the first steps in arterial plaque formation [62]. It is believed that circulating ceramides may also promote oxidized lipoprotein infiltration into the vessel wall, thus facilitating monocyte adhesion to the vessel wall, atherosclerotic plaque formation, and expansion of the plaque, as well as promoting plaque rupture [63,64,65,66].

We and our associates at the “Thrombosis Research” laboratory, Lillehei Heart Institute, University of Minnesota, discovered that glutathione in platelets stored under laboratory conditions undergo circadian rhythm [67]. It is well known that oxidative stress is one of the causes for the reduced efficacy and shelf life of platelets [63]. Our research team explored the dose-dependent effect of chemical oxidizing agents such as N-ethylmaleimide (NEM), as well as 1-chloro-2,4-dinitrobenzene on platelet glutathione (GSH) levels. Arachidonic acid (AA) induced aggregation and secretion responses in platelets depleted of GSH, occurred at lower doses of agonists, suggesting that GSH-depleted platelets were hypersensitive to the action of these agonists. Despite the lower production of the lipoxygenase products of AA, significant 12-HETE production was observed by GSH-depleted platelets, suggesting that GSH and GSH-Peroxidase are not required for the generation of 12-HETE in human platelets [63]. These studies suggest that the three modes of treatment share a common mechanism of increasing AA metabolism to biologically active prostaglandin formation through alterations in cyclooxygenase kinetics and enzyme availability [67,68,69,70,71,72]. Despite these observations, studies have shown that physiologically relevant concentrations of HPETEs potentiate platelet aggregation, mediated via a stimulation of cyclooxygenase activity [65]. Although platelets have been implicated in inflammation, the role and mechanisms of specialized pro-resolving mediators (SPMs), such as oxylipins, produced by immune cells in platelet function are not clear [73].

## 5. Endothelial Dysfunction and Inflammation

**Figure 2:**
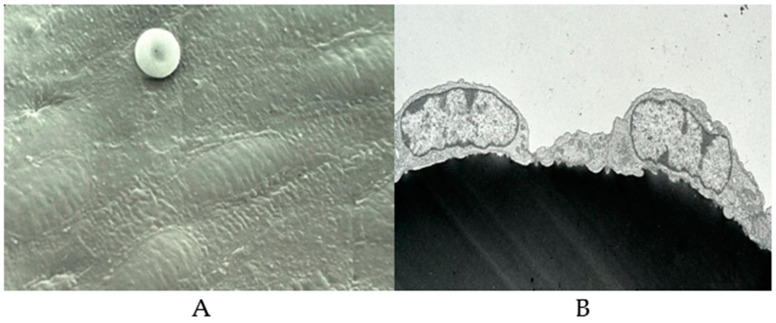
Scanning electron micrographs of healthy endothelium and endothelial cells. [Courtesy: (Late) Professor James G White, University of Minnesota.] (**A**) Healthy endothelium. (**B**) Two endothelial cells held together.

The blood vessel wall consists of three layers; intima, media, and adventia [74]. The intima consists of endothelium and subendothelial tissues and is separated from the media by the elastic lamina interna. Endothelial cells form a continuous monolayer, lining all blood vessels and acting as a barrier between the circulating blood products and the extracellular matrix (ECM) components (Figure 2). The blood vessel wall is covered with over a trillion endothelial cells (ECs), whereas roughly 150 × 10^9^/ L to 400 × 10^9^/L of platelets circulate in these blood vessels. Platelets are in continuous contact with the surface of the blood vessels and upon any injury immediately jump into action to arrest bleeding and initiate the wound-healing process. Lining the entire surface of blood vessels, endothelial cells provide in the steady state an anti-inflammatory, anticoagulatory surface. However, in the case of an injury or infection, endothelial cells control the adhesion and migration of inflammatory cells, as well as the exchange of fluid from the bloodstream into the damaged tissues.

**Figure 3:**
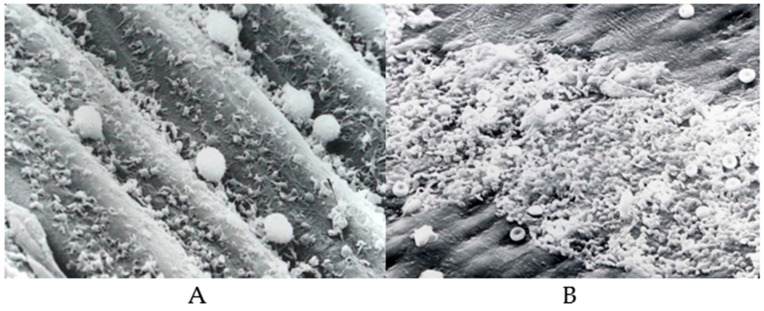
Scanning electron micrographs of platelet interaction with endothelium. [Courtesy of (Late) Professor James G. White, University of Minnesota.] (**A**) Dysfunctional endothelium. (**B**) Damaged endothelium.

In cases of infection or any kind of surface injury, endothelial cells undergo morphological and functional modifications [68]. Endothelial dysfunction is the result of an imbalance between vasodilators and vasoconstrictors produced by circulating platelets and vessel wall enzymes. Some of the vasoactive compounds released by the ECs include vasodilatory compounds such as adenosine, prostacyclin, and nitric oxide and vasoconstrictory molecules, such as cyclooxygenase-derived endothelium dependent constriction factor (EDCF), hypoxia-induced endothelium dependent constriction factor (EDCF), and endothelin, a vasoconstrictor [75,76,77,78], whereas lipid peroxides, oxidized lipids, and lipoproteins and various metabolic disease states promote the formation of vasoconstrictors. Shown in Figure 3 are typical platelet interactions on dysfunctional endothelium (Figure 3A) and damaged endothelium (Figure 3B).

As markers of endothelial function/dysfunction, adhesion molecules, selectins, prothrombotic molecules, and inflammatory molecules have been used. In a first of a kind clinical study, researchers examined the effects of anti-inflammatory agents such as methotrexate (MTX), low-dose colchicine, and their combination in stable CVD patients. Serum markers of inflammation and systemic endothelial function measures were not significantly different among the patient and control subjects [75]. According to some researchers, anti-inflammatory molecules and antioxidants seem to improve endothelial dysfunction, independently from their lipid-lowering property [76,77,78]. Studies from our laboratory demonstrated that known anti-platelet drugs including non-steroidal anti-inflammatory agents do not inhibit platelet interactions with exposed sub endothelium or with extra cellular matrix components [79]. What we are missing here is the lack of a specific marker for inflammation and a specific treatment that suppresses the inflammation, as measured by inflammatory markers. For instance, Crohn’s disease (CD) is a chronic inflammatory disease that causes inflammation of the digestive tract. It belongs to the larger group of illnesses called inflammatory bowel disease (IBD). The major inflammatory agent seems to be TNF-α. The broadly recognized biomarker of IBD is fecal calprotectin (FC) and fecal lactoferrin (FL). Fecal miRNAs, miR-16, miR-21, miR-155, and miR-223 show differential expression levels in IBD [71]. Infliximab is an IgG1 (murine) and human (75%) chimeric monoclonal antibody (mAB), targeted against TNF-α. A study has documented the efficacy of this agent in the induction and maintenance of response and remission in treatment-refractory inflammatory CD. When it comes to inflammatory disease in general and IBDs in particular etiopathologies remain poorly understood. A better understanding of the immunological mechanisms involved in IBDs/CDs is critical to the development of novel therapeutic interventions [80,81].

## 6. Atherosclerosis and Inflammation

**Figure 4:**
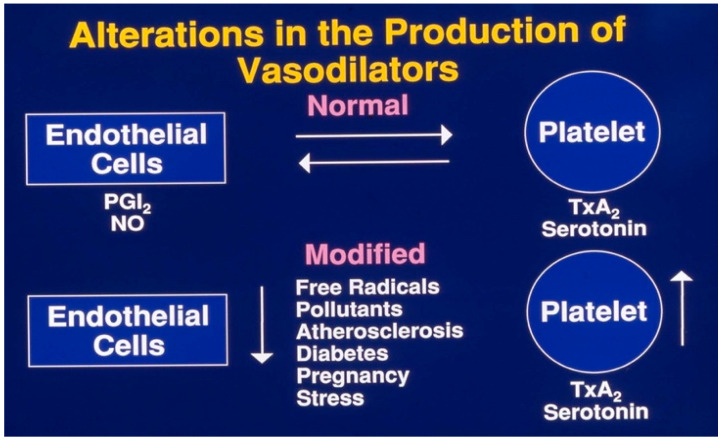
Schematic presentation of altered production of vasoactive molecules. (University of Minnesota, Artists. Personal Collection).

As we have discussed earlier, the initial event in the pathogenesis of atherosclerosis is injury to the endothelium (Figure 3B and Figure 4). In response to injury, the first responders adhere to the site of injury and recruit platelets, leucocytes, and primarily monocytes, resulting in the accumulation of lipids, particularly oxidized lipids, and lipoproteins (LDL), and the transformation of monocytes to macrophages that ingest lipids and promote inflammation and the development of fatty streaks. Atherosclerosis is considered a chronic inflammatory disease [82]. Chronic inflammation seems to display four characteristic features, lympho-monocytic infiltration, sclerosis, cellular proliferation, and vascular proliferation; all of these features occur during the development of atherosclerosis. Riksen’s team analyzed bone marrow samples from 13 individuals with severe atherosclerosis and found that some inflammatory genes were active in the monocyte precursor stem cells of these 13 people, than in those of healthy individuals [83]. Some of the compounds that show promise for blocking trained immunity include inhibitors that reduce interleukin 1β, a cytokine known to have a role in cardiovascular disease [84,85,86,87].

Altered lipid metabolism seems to play an important role in the development and progression of atherosclerosis [82]. However, lipid lowering does not eliminate the risk of developing the disease. Furthermore, cardiovascular disease is more common in people with chronic inflammatory diseases such as rheumatoid arthritis and chronic infection of the gums. Researchers have shown that pathogens, stress hormones, and cholesterol can all train immune cells, which are known to play a major part in atherosclerosis. When monocytes encounter an atherosclerotic plaque on the vessel wall, they morph into macrophages and release inflammation-inciting molecules called cytokines. Macrophages also release enzymes that eat away at protein and cause plaque destabilization.

**Figure 5:**
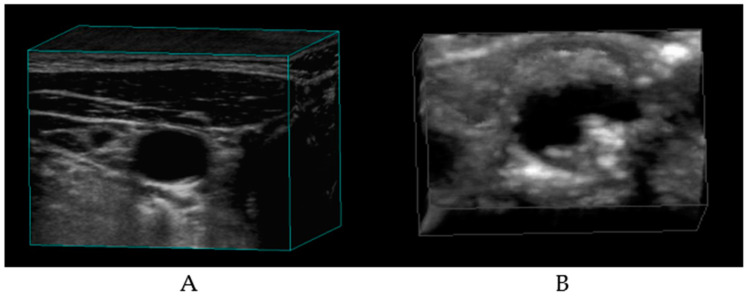
Three-dimensional ultrasound images of carotid arteries. (Courtesy: Dr Aaron Fenster, Research Director, Robarts Research Institute, Canada.) (**A**) Carotid artery with minimal stenosis. (**B**) Carotid artery with plaque.

Keener, in an article in *Scientific American* dated November 2021, makes a case for innate immune cells as treatment targets for atherosclerosis [84]. The CANTOS trial was a double-blind, randomized study investigating the effects of canakinumab, a monoclonal antibody against the proinflammatory cytokine IL-1β, in patients with recent myocardial infarction (MI). The 150 mg canakinumab dose led to significantly fewer recurrent cardiovascular events [84,85,86,87]. It should be noted that the observations in these studies suggest a lowering of acute arterial events, not necessarily a reduction or reversal of atherosclerosis, whereas in a seminal study, Professor J D Spence of Robarts Research Institute, Western University, Ontario, Canada, demonstrated a case in which the plaque volume of a patient increased from 20 mm^2^ to 28 mm^2^ after stopping treatment with rosuvastatin. After resuming statin (5 Mg/d), the plaque area regressed to 19 mm^2^ in just two months, demonstrating the effect of lipid-lowering agents on the plaque volume in carotid arteries [88,89]. These investigators used 3D ultrasound measurements to determine the plaque volume (Figure 5).

Using a high-duplex ultrasound machine, they obtained carotid scans. They used proprietary software analytics to obtain 3D ultrasound images of the carotid artery. In recent years, they have been using deep learning techniques to improve automated measurements of plaque burden. Based on the results of their studies, they developed a concept of treating arterial lesions rather than the management of risk factors for stroke [89]. We are also of the opinion that balancing the altered metabolism is better than the management of risks for metabolic diseases.

Dr Ross, a distinguished Professor of Pathology, University of Washington, Seattle, described atherosclerosis as in inflammatory disease more than two decades ago [82]. Several arguments, both pros and cons, have appeared since then about this hypothesis. Just like the famous Barker hypothesis of the “fetal origin of adult diseases” oxidized low-density lipoprotein (LDL) is widely distributed in the fetal aorta, but atherosclerotic plaques do not appear until later in adult life (third or fourth decade). Furthermore, just the presence of foamy macrophages in a fatty streak does not make the lesion inflammatory. This is a very important and debatable part of the sequalae of CVD risk factors. The prestigious *Journal of Biomolecules* has even published several special issues on the topic of atherosclerosis [90,91,92]. Readers are urged to refer to comprehensive reviews on this topic to understand the complexities of this multifactorial arterial lesion.

## 7. Hypertension and Inflammation

Incidence and prevalence of hypertension has rapidly increased to epidemic proportions and is leading cause of premature death worldwide [93,94,95,96,97,98,99,100]. It is very well known that important modifiable independent risk factors for premature mortality include the development of endothelial dysfunction, atherosclerosis, and arterial vascular diseases. Recent studies have demonstrated a role for sodium-induced immune cell activation in the pathogenesis of hypertension by the modulation of the gut microbiome and the generation of lipid oxidation products known as isolevuglandins (IsoLG) [96]. They are also known to cause the elaboration of inflammasomes, leading to end-organ failures. Furthermore, they may also promote the proliferation of some immune cell populations. In brief, high salt concentrations can cause immune cells to produce cytokines that promote inflammation and organ damage. Immune cells such as macrophages and T cells are found in sodium-rich intestinal microenvironments. The ubiquitous poly unsaturated fatty acid arachidonic acid (AA) is metabolized under normal conditions to vasoactive metabolites by vascular tissues and circulating platelets. In addition, in certain situations AA reacts with oxygen and forms peroxyl radicals, which promote the formation of lipid aldehydes such as malondialdehyde (MDA), 4-hydroxy-nonetal (HNE), 4-oxo-nonetal methylglyoxal (MGO), and isolevuglandins. Any tissue with significant AA content is prone to oxidative damage and will likely produce elevated levels of IsoLG adducts [96]. These bioactive molecules seem to drive neutrophil migration in hypertension and also promote neutrophil extracellular trap (NET) formation [97]. NETs have been implicated in the pathogenesis of CVDs via mechanisms that promote endothelial cell injury, activation, inflammation, and thrombosis.

Highly reactive metabolites of AA, isolevuglandins, have been detected in many diseases linked to oxidative stress. In the 1980s, we at the University of Minnesota conducted extensive studies on the role of free radicals, peroxide tone, and heme-AA interaction, as well as oxidative stress, on arachidonic acid metabolism and platelet function [67,69,70,71,98]. Since our focus was on the vasoactive metabolites of AA, we did not find this important metabolite of AA. Cardiologists at Vanderbilt University have described these highly reactive bioactive lipids as key players in cardiovascular disease [99]. According to these researchers, isoLG adducts are elevated in multiple diseases linked to inflammation and oxidative stress, including hypertension, obesity, atherosclerosis, and Alzheimer’s disease [99]. By preemptively scavenging isoLG, a small molecule, 2-hydorxybenzylamine, a natural compound found in buckwheat, reacts with isolevuglandins to prevent the formation of isoLG adducts with proteins and DNA. Chinese cardiologists have suggested that hypertension, through vasoactive peptides such as angiotensin and endothelin-1, promotes and accelerates the atherosclerotic process via inflammatory mechanisms [100]. According to these researchers, angiotensin-11 (Ang-11) promotes the generation of reactive oxygen species in the endothelium, smooth muscle cells, and the adventia of blood vessels, leading to endothelial dysfunction, inflammation, the upregulation of endothelin-1, adhesion molecules, NF-kappa B, and other inflammatory mediators.

Because of these observations, inflammation is considered a bridge between hypertension and atherosclerosis (100). Ang-11 interrupts the anti-inflammatory role of insulin, whereas Ang-11 blockers suppress mediators of inflammation, including ROS and C-reactive protein (CRP), and they increase the expression of inhibitory NF-kappa B. Based on the results of such studies, the authors conclude that “such anti-inflammatory and antioxidative effects may be in part due to unopposed stimulation of the Ang-11 type receptor blocker and therefore, may be beneficial in the management of acute coronary syndromes and may also contribute to the prevention of type-2 diabetes mellitus, as insulin resistance is mediated by inflammatory processes” [101]. Chronic inflammation, therefore, is mediated by various modulators, including Ang-11, proinflammatory cytokines, free fatty acids, and their highly reactive derivatives. Therefore, to be effective, one will have to develop combination therapy that addresses multiple mechanisms that drive the progression of chronic occlusive arterial vascular diseases (COAVD). Despite such efforts still question remains as to whether the reversal of oxidative stress and inflammation provides total vascular protection, as we are dealing with just two of the multiple factors that contribute to the progression of these diseases [102].

## 8. Obesity and Inflammation

Excess weight and obesity, similar to hypertension, have reached epidemic proportions and affect over 1.9 billion people worldwide [103]. Metabolic diseases in general, as well as the risk factors that initiate and promote these risks, share a common chronic inflammatory condition. As far as excess weight and obesity are concerned, signaling via cytokines of the TNF family seems to mediate cell death and inflammation of the adipose tissue, resulting in lipid spillover, glucose toxicity, and insulin resistance [104]. The sequalae of events leading from obesity to inflammation seem to be that obesity induces cell death and inflammation of the white adipose tissue (WAT), leading to the chronic inflammation associated with metabolic diseases. Mechanisms involved in the death of WAT and Nf-κB-mediated inflammation regulate energy homeostasis and insulin sensitivity. Individuals with excess weight have altered TNF-α, C-reactive protein, interleukins (IL-6, IL-8), resistin, and visfatin [103,104,105,106,107].

The usual treatments for the management of obesity include lifestyle changes, recommendation of dietary changes, and where needed surgical interventions and pharmacotherapy. Conventional treatments for the management of obesity and diabetes, including metformin GLP1 receptor agonists and TZDs, lead to improvements in insulin sensitivity and provide anti-inflammatory benefits. On the other hand, anti-inflammatory therapies often fail to improve, and frequently worsen, insulin sensitivity [107]. Although several studies on the use of anti-inflammatory agents for obesity-related inflammatory conditions are reported, the lack of effective therapeutics indicates the need to assess molecular mechanisms associated with obesity and inflammation. Readers are urged to refer to comprehensive reviews on this topic [1,2,3,4,5,6,7,8,9].

## 9. Type-2 Diabetes and Inflammation

According to the global burden of diseases, between 1980 and 2021, the number of adults with diabetes (type-2) increased from 108 million to 537 million, with corresponding increases in obesity from 100 million to 754 million adults. No nation has experienced a decline in diabetes or obesity in the last several decades [108]. Experts are of the opinion that components of the immune system are altered in obesity and type-2 diabetic patients. Most changes seem to occur in the adipose tissue, the liver, pancreatic islet cells, the vasculature, and leukocytes [109]. However, glucose is the major source of energy for most of the tissues, and glucose homeostasis is maintained by complex regulatory mechanisms. Altered glucose metabolism and increases in blood glucose levels induce oxidative stress in patients with type-2 diabetes. The presence of high levels of biomarkers such as 4-hydorxy-2-nonetal proteins, 8-hydroxy-deoxyguanosine, and 8-epi-prostagladin F_2_α in the serum of diabetic patients has been reported [100]. Furthermore, in diabetics, the levels of cholesterol, triglycerides, and free fatty acids are also increased, thus adding the burden of lipotoxicity [110]. The prolonged expression of pancreatic beta cells has been reported to inhibit insulin gene expression. These two types of toxicities occur independently. There is considerable interest in the use of antioxidant drugs as protectors against beta cell oxidative stress.

Insulin signaling mechanisms are complex, yet in terms of its role in diabetes, the focus will be on its role in glucose metabolism in target tissues such as the liver, adipose tissue, and skeletal muscles [111,112]. The two well-known pathways are the phosphatidylinositol 3-kinase (PI3K)-AKT pathway, which is largely responsible for insulin’s action on glucose uptake. The second pathway is the Ras-mitogen-activated protein kinase (MAPK) pathway, which mediates gene expression, as well as participating in cell growth and differentiation. Stimulation of the NF-κB and AP-1 Fos/Jun inflammatory pathways results in the activation of serine kinases, Iκκb, and Jnk1. A study has reported chronic activation of proinflammatory signaling pathways, leading to increased levels of TNF, IL-6, interleukin (IL-8), CRP, and decreased insulin sensitivity [113]. Insulin suppresses proinflammatory transcription factors in mononuclear cells and thereby reduces subsequent inflammatory mediators [114]. Anti-diabetic drugs such as pioglitazone, rosiglitazone, troglitazone, metformin, sulfonylureas, and GLP-1 receptor antagonists seem to modulate inflammatory activity. Based on the analysis of 16 randomized clinical trials (RCTs), researchers from China concluded that “anti-inflammatory therapies targeting pathogenic process of diabetes can significantly reduce the level of fasting glucose, HbA1c, and CRP in patients with diabetes” [115]. These researchers also studied the effects of anti-inflammatory therapies on different inflammatory targets, including IL-1β, IL-1βR, TNF-α, and NF-κB. Their findings were similar to the findings of the RCTs.

## 10. Arterial Occlusive Events and Inflammation

The major arteries that develop atherosclerosis and stenosis are the arteries of the limbs, heart, and brain as well as small vessels of the cerebral vascular tree [116]. In fact, cerebral small vessel disease is responsible for 25% of all strokes. Vascular diseases are ranked the number one killers and heart disease is likely to remain the number one killer due to the long-term COVID-19 impact. According to the World Heart Federation, Geneva, deaths from CVDs surged 60% globally over the last three decades and jumped worldwide, from 12.1 million in 1990 to 20.5 million in 2021. Inflammation seems to accompany all phases of the sequalae of risks that promote the development of metabolic diseases as well as thrombotic events. Therefore, understanding the role of inflammation in arterial occlusive events is crucial for developing effective preventive and treatment strategies. By targeting inflammation, it may be possible to reduce the risk of atherosclerosis and its associated complications. However, further research is still needed to fully understand the complex interplay between inflammation and arterial occlusion.

**Figure 6:**
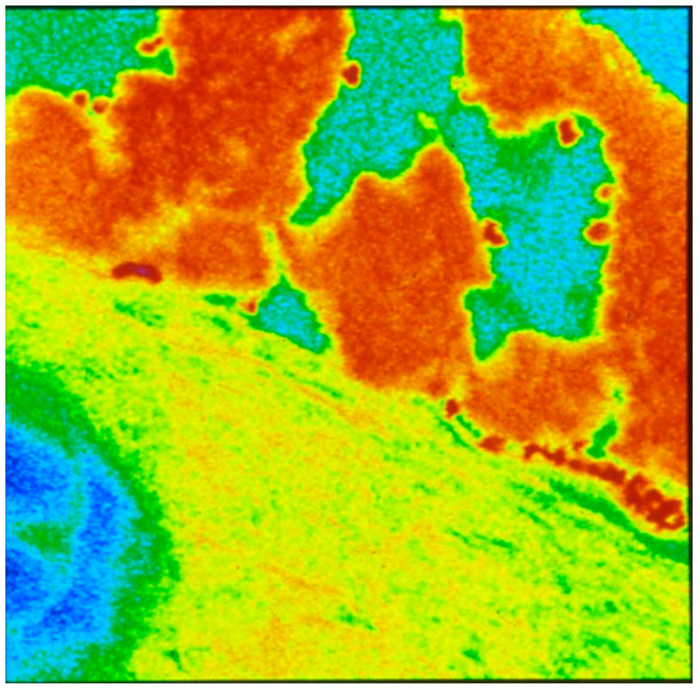
Thrombus formation on denuded endothelium. (Personal Collection).

Figure 6 shows platelets’ interaction with the denuded subendothelium of rabbit aorta. In this study, denuded rabbit aorta was used for demonstrating the platelet-rich thrombus formation during the circulation of human blood over the denuded surface. The tissues were fixed post exposure and sections were cut and stained for assembled actin, which is the major component of the platelet-rich thrombus. Diabetic subjects have hyper aggregable platelets. “Diabetic platelets” are characterized by the dysregulation of several activation signaling pathways, leading to persistent in vivo platelet activation, induced by hyperglycemia, insulin resistance, inflammation, oxidative stress, and endothelial dysfunction [117,118,119,120,121]. The central role of platelet activation in acute coronary syndrome is further supported by increased prostaglandin metabolites detected in patients with acute coronary syndromes, as well as the clear clinical benefit of aspirin for the prevention of acute coronary events [121,122,123,124].

**Figure 7:**
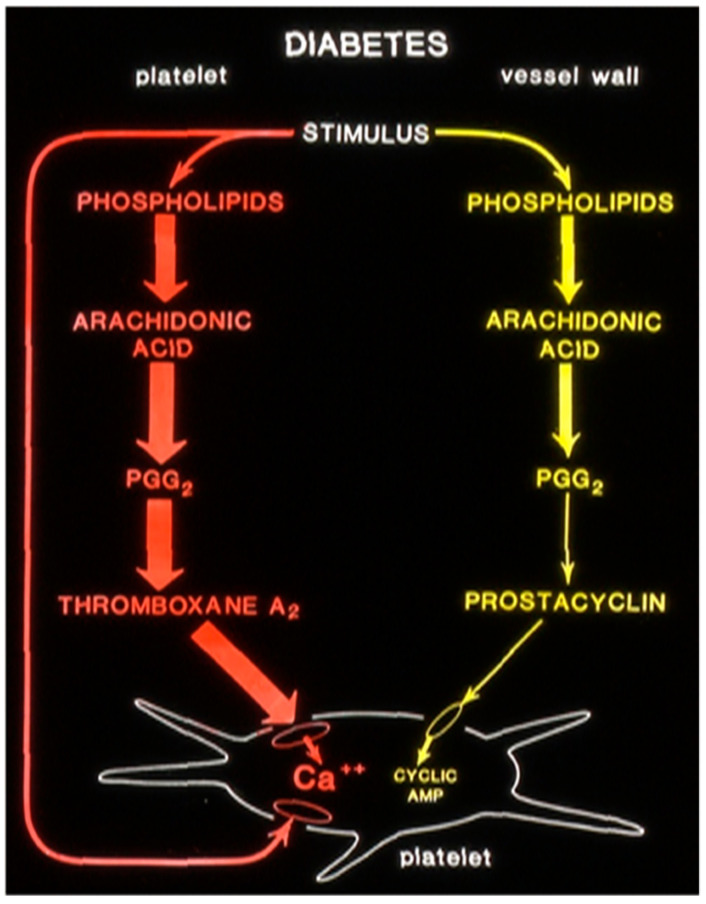
Altered prostaglandin metabolism in drug-induced diabetic rats. (Courtesy: Dr Jonathan Gerrard, Professor University of Winnipeg, Canada).

In a one-of-a-kind study at the University of Minnesota, we in collaboration with Dr Gerrard demonstrated altered arachidonic acid metabolism in drug-induced diabetic rats. In these studies, the rats were made diabetic by an injection of streptozotocin. We measured prostanoids’ production by platelets and vascular tissues by measuring the stable metabolites of radiolabeled arachidonic acid, thromboxane, and prostacyclin [124]. In diabetic rats, the thromboxane production was increased and prostacyclin production was decreased compared to that of control animals (Figure 7). Upon the transplantation of pancreatic islet cells to diabetic rats, the prostaglandin production was normalized, showing the alterations in prostanoids’ production favoring a thrombotic condition were disease-specific.

## 11. Discussion

Inflammation is the first responder when there is tissue injury [125,126,127,128,129,130]. There are basically three mechanisms involved in wound healing: inflammation, proliferation, and remodeling. In the acute phase, it involves activation of the platelet and coagulation pathways, so that bleeding is arrested, followed by the release of chemicals that cause blood vessels to dilate and become more permeable, allowing immune cells to enter the wound site. During the next phase, new tissues are generated to fill the wound and restore its structure and function. The final phase is remodeling, where the newly formed tissue undergoes maturation and remodeling.

Several traditional risk factors for CVD, such as hypertension, hypercholesterolemia, and diabetes, have been associated with inflammation [99,100,104,106,109,113]. Chronic inflammation can lead to endothelial dysfunction, which increases the permeability of lipoproteins and their accumulation in the arterial wall. This promotes the recruitment of leukocytes and activation of platelets, further contributing to the inflammatory process. Macrophages, derived from monocytes, secrete inflammatory cytokines that contribute to the progression of atherosclerosis. Oxidized lipoproteins and triglyceride-rich lipoproteins have proinflammatory effects, and chronic psychological stress can also increase the risk of CVD through inflammation-mediated pathways. Inflammation serves as a link between aging and CVD due to increased clones of leukocytes in the peripheral blood. Managing inflammation is crucial in reducing the risk of CVD. Anti-inflammatory interventions that target cytokine pathways have shown potential in reducing the risk of recurrent myocardial infarction and stroke. However, it is important to note that these interventions may also increase the risk of infections.

Having discussed the role of inflammation in health and disease briefly, we need to ask a serious question. Is the inflammation a cause or a bystander in most of the progression of metabolic risks. According to experts, inflammation is considered more than a bystander. It can be both a cause and a consequence of various metabolic disorders, starting from oxidative stress, which is hard to detect and treat. In response to metabolic stress, both the innate and adaptive immune system become activated during metabolic dysfunction-associated steatotic liver disease (MASH). Understanding the interaction between immune cells and parenchymal cells seems to be the key to deciphering the pathogenesis of MASH. There seems to be important link between stress and metabolic diseases, such as metabolic syndrome, and vascular diseases, type-2 diabetes, heart attack, and stroke [125,126,127,128,129,130]. Furthermore, obesity, which is associated with metabolic risks, is characterized by low-grade chronic inflammation. Such a chronic inflammatory state contributes significantly to the development of different metabolic disorders, including insulin resistance. Inflammation and cell death are noticed during obesity, and they are closely associated with metabolic dysregulation [94,111]. Therefore, there are suggestions that inflammation should be considered an active participant of metabolic disorders and further exacerbate their progression.

The overall evidence base of the benefits of anti-inflammatory therapies in reducing, reversing, or preventing metabolic diseases or acute vascular events has not been systematically evaluated [114]. Researchers from the National Clinical Research Center for Metabolic Disease, Hunan, China, studied 16 random controlled clinical trials comprising 3729 subjects in the metanalyses and found that anti-inflammatory therapies targeting the pathogenic process of diabetes can significantly reduce the level of fasting plasma glucose (FPG), HbA1c, and CRP in patients with type-2 diabetes [115]. These researchers concluded “that targeting cytokines, cytokine receptors, and inflammation-associated nuclear transcription factors, such as IL-1β, IL-αR, TNF-α, and NF-kB alone or in combination, can significantly reduce the level of FPG, HbA1c, and CRP in patients with type-2 diabetes. Despite these findings, there are no such recommendations by any of the major professional societies or healthcare providers, because clinically approved drugs are available for reducing these contributing factors. Furthermore, these therapies more or less manage the modifiable risk factors and do not reverse or prevent type-2 diabetes.

The proinflammatory cytokine interleukin-1β plays multiple roles in the development of atherosclerotic plaques, including the induction of procoagulant activity, the promotion of monocyte and leukocyte adhesion to vascular endothelial cells, and the growth of smooth muscle cells. Paul Ridker and associates from the Center of Cardiovascular Disease Prevention, Brigham and Wolens’s Hospital, Boston Mass., conducted the CANTOS Trial in which canakinumab, an anti-inflammatory drug, was tested in a randomized trial of 10,061 patients with a previous myocardial infarction [87]. In this study, they found that the drug reduced CRP significantly and lowered the rate of recurrent cardiovascular events. The researchers suggest that targeting inflammation with canakinumab may reduce the risk of cardiovascular events. CRP is a nonspecific marker of inflammation and has an important value in the management of various inflammatory and immunomodulatory states, including microbial infections, trauma, tissue damage, and neuro-degenerative conditions. CRP is a well-established and stable marker of chronic low-grade inflammation, but it is not thought to be part of the causal pathway between inflammation and CVD itself [127,128]. Therefore, a reduction in CRP would not necessarily mean equivalent reduction in metabolic disease risks or CVD events.

Researchers from the Clinic of Endocrinology, Diabetes Metabolism and Department of Biomedicine, University of Basel, Switzerland, concluded that “alternative anti-inflammatory treatments, alone or in combination, may turn out to be more effective, depending on genetic predispositions and the duration and manifestation of the disease. Thus, there is a great need for comprehensive and well-designed clinical studies, to implement anti-inflammatory drugs in the treatment of patients with metabolic syndrome and associated conditions” [130]. In summary, inflammation plays a significant role in the development and progression of cardiovascular disease risk factors. Understanding and managing inflammation are crucial for reducing the risk of developing CVD and its associated complications. When discussing the role of inflammation in cardiometabolic diseases, the challenges are determining how metabolic inflammation starts and when this process becomes detrimental to the tissues and organ systems [131]. Several mechanisms have been proposed including the release of toxic compounds from dying adipose tissues. However, it is not clear at this time which specific mechanisms trigger these events. Further research is needed to explore the specific molecular mechanisms involved in the inflammatory process and develop targeted anti-inflammatory therapies.

## 12. Conclusions

In conclusion, inflammation occupies a central role in the genesis and progression of risk factors for cardiometabolic diseases. A thorough understanding and managing inflammation are critical components of mitigating the risk of developing cardiometabolic diseases and associated complications. Further research is needed to unravel the precise molecular mechanisms underlying inflammation and to develop robust, specific, and effective anti-inflammatory therapies. The quest for alternative anti-inflammatory treatments and their potential effectiveness, especially considering genetic dispositions and the duration and manifestation of diseases like cardiometabolic diseases, underscores the necessity for well-designed randomized clinical studies.

## Data Availability

Not applicable.
